# Ten Years of London Pauperism

**Published:** 1903-09-12

**Authors:** 


### HOSPITAL ADMINISTRATION.

### CONSTRUCTION AND ECONOMICS.

### TEN YEARS OF LONDON PAUPERISM.

In 1901 the rate-payers of the Metropolis were paying two shillings in the pound for poor relief, and the rate had been steadily rising for years past. It may be, as optimists assure those who venture to grumble at the burden, that the wealth of the country as a whole has so increased that the increasing rates are not severely felt. But if the wealth of the country is increasing, why is pauperism not diminishing? This was the conundrum put by Sir William Chance at a recent meeting of the Royal Statistical Society, and though he offered no definite solution of the general rise in rates, he was able to show that the administration, as well as the actual poverty, of a district had a clear effect on the increased expense. For it is not always the poorest districts which have the largest proportion of paupers. Thus, it is putting the case very mildly to say that St. George's, Hanover Square, is not such a poor district as Bethnal Green, yet the percentage of paupers is higher in the former than in the latter—21·9 per thousand as against 25·7—although the population is diminishing, and the decrease is among the poorer classes, several slums having been cleared away during the last ten years. This last is a point which must always be taken into consideration, for it is quite possible for pauperism to decrease in a district simply through the places where the poor lived being cleared away, and the denizens compelled to take themselves and their poverty to some less favoured neighbourhood. But St. George's absolutely shows such a change in the character of the population as should cause pauperism to diminish, and therewith an increase.

Even in cases where the social condition of neighbouring parishes is much the same, there are great differences in the number of paupers. Thus there are 9·5 paupers to every thousand people in Paddington, while in Kensington the proportion is 15·2. The parishes of St. Olave's and St. Saviour's (Southwark) have much in common, yet St. Saviour's has 24·3 paupers to the thousand, while St. Olave's has 49, and is in the position of having the highest proportion in London. Lambeth and Camberwell have a similar class of inhabitants, and in 1891 pauperism was greater in the former, but while its rate has fallen from 2·29 per cent. of population to 1·76, Camberwell has risen from 2·10 to 2·60. That being so, it is not strange that the poor-rate has also risen, and that while in 1891 the rates in Camberwell were 1¾d. less than in Lambeth, the position in 1901 had changed so much that Camberwell paid 8d. in the pound more.

The inference is that pauperism does not depend so much on the poverty of a district as on its administration. This is certainly not an absolute rule, for some districts will always have more poor than others—we do not require to be told that Hampstead will have fewer paupers than Whitechapel—but large differences in the amount of pauperism in districts in most ways similarly situated point to the fact that in some there is much greater leniency than in others.

Leniency begins in outdoor relief. That is almost a fixed rule. Many people who would—at least at the beginning of their career of pauperism—refuse to go to the workhouse, are quite willing to take half-a-crown a week from the parish, and their relatives, who would not let them enter "the house," will agitate to get them this dole. That is, to begin with; but the receipt of charity blunts the feelings of the recipient, and in the end indoor relief is not disdained Thus it is that we find that in most cases outdoor pauperism and indoor increase together. In the ten years with which Sir William Chance dealt he showed that of thirteen unions which had increased their total pauperism, eleven had increased their outdoor pauperism. Very rarely does it appear that an increase of outdoor relief lessens the sum of indoor pauperism. As Sir William Chance put it, "a lax policy of outdoor relief so demoralises a population, by leading them to look to the State instead of to their own efforts for support and help in times of difficulty, as to reduce it by degrees to such a state of indigence and idleness that, *nolens volens*, only indoor relief can be given."

It should be said, however, that a part of the outdoor relief is medical relief only. It is a question if this can or should be stopped. Very often those who can maintain themselves in health cannot do so when sickness enters the household. But while that may be admitted, it is undeniable that in this department also much abuse exists. The parish of St. Pancras managed, during the two last years of the decade, to reduce the grants of medical relief by 46 per cent., by merely insisting on the applicants attending before the relief committee. It would be interesting to know how many actually went before the committee and were refused, and how many would not take the trouble of attending at all. Indoor medical relief accounts for a large increase both in the numbers of the indoor poor and of the expense of relieving them. But this is almost wholly a good thing. Even as late as 1891, workhouse infirmaries left much to be desired. In almost every point they were inferior to the voluntary hospitals. They still suffer, as all compulsory institutions do, from a lack of public interest in them. They are less cheerful than hospitals, and not so well provided with those amenities which often make a stay in hospital a refining influence in the life of a patient. Still it may now be said that, in the metropolitan infirmaries, the patients are well looked after and properly nursed, and in the present condition of the voluntary system they cannot be dispensed with. There has also been a great improvement in the matter of isolation hospitals, the condition of which both as regards accommodation and administration formerly often deserved the severest criticism. The growing regard for public health has led to a determination to isolate all infectious cases where it cannot be shown that there is adequate provision for nursing them at home, and this has led to a great but justifiable expense, of which the full benefit cannot yet be known. Some people might protest against the steadily increasing size of the salary list. In 1871, before poor-law matters had aroused much public interest, the proportionate expense of salaries compared with the total expenditure was not 10 per cent., now it is 23. It certainly seems, at the first glance, a thing to be regretted that nearly a quarter of the money raised by the rates should go in administration expenditure, but there can be no doubt that there has been a great increase in the efficiency of all poor-law officials of late years. This is especially true of those who have to deal with the sick, but in all departments there is shown more care in dealing with cases, and a more determined attempt to prevent the development of a race of hereditary paupers. If the employment of a greater number of officials and paying them higher salaries, produced this result, the increase in that item needs no apology.

